# Migraine with Aura Is Associated with an Incomplete Circle of Willis: Results of a Prospective Observational Study

**DOI:** 10.1371/journal.pone.0071007

**Published:** 2013-07-26

**Authors:** Brett Cucchiara, Ronald L. Wolf, Lidia Nagae, Quan Zhang, Scott Kasner, Ritobrato Datta, Geoffrey K. Aguirre, John A. Detre

**Affiliations:** 1 Department of Neurology, University of Pennsylvania, Philadelphia, Pennsylvania, United States of America; 2 Department of Radiology, University of Pennsylvania, Philadelphia, Pennsylvania, United States of America; 3 Department of Radiology, Children's Hospital of Philadelphia, Philadelphia, Pennsylvania, United States of America; 4 Department of Radiology, Tianjin Medical University, Tianjin, China; University Medical Center Goettingen, Germany

## Abstract

**Objective:**

To compare the prevalence of an incomplete circle of Willis in patients with migraine with aura, migraine without aura, and control subjects, and correlate circle of Willis variations with alterations in cerebral perfusion.

**Methods:**

Migraine with aura, migraine without aura, and control subjects were prospectively enrolled in a 1∶1∶1 ratio. Magnetic resonance angiography was performed to examine circle of Willis anatomy and arterial spin labeled perfusion magnetic resonance imaging to measure cerebral blood flow. A standardized template rating system was used to categorize circle of Willis variants. The primary pre-specified outcome measure was the frequency of an incomplete circle of Willis. The association between circle of Willis variations and cerebral blood flow was also analyzed.

**Results:**

170 subjects were enrolled (56 migraine with aura, 61 migraine without aura, 53 controls). An incomplete circle of Willis was significantly more common in the migraine with aura compared to control group (73% vs. 51%, p = 0.02), with a similar trend for the migraine without aura group (67% vs. 51%, p = 0.08). Using a quantitative score of the burden of circle of Willis variants, migraine with aura subjects had a higher burden of variants than controls (p = 0.02). Compared to those with a complete circle, subjects with an incomplete circle had greater asymmetry in hemispheric cerebral blood flow (p = 0.05). Specific posterior cerebral artery variants were associated with greater asymmetries of blood flow in the posterior cerebral artery territory.

**Conclusions:**

An incomplete circle of Willis is more common in migraine with aura subjects than controls, and is associated with alterations in cerebral blood flow.

## Introduction

An estimated 28 million Americans suffer from disabling migraines. Beyond the immediate disability related to headache, migraine, particularly with aura, has also been associated with an increased risk of ischemic stroke [1], [2], [3], [4], [5], [6], [7]. Mechanistically, cortical spreading depression (CSD) appears to play a critical role in migraine with aura, and possibly migraine without aura [8]. While the trigger for initiation of CSD in migraine remains incompletely understood, some evidence suggests that alterations in cerebral blood flow may trigger both migraine and CSD [9], [10].

Within this framework, one factor not previously well investigated is the contribution of structural alterations in the cerebral vasculature, specifically variations in the circle of Willis, to migraine pathogenesis. The circle of Willis serves as a source of collateral flow to maintain brain perfusion, and has highly variable anatomy, with only 40–50% of normal subjects having an entirely complete circle [11]. Circle of Willis variations have been associated with alterations in intracranial vessel blood flow volume and regional cerebral blood flow (CBF) [12], [13]. To investigate the hypothesis that an incomplete circle of Willis is associated with migraine, we performed the Anatomy and Cerebral Hemodynamic Evaluation of Migraine (ACHE-M) study, using high-resolution magnetic resonance angiography (MRA) to evaluate circle of Willis structure and arterial spin labeled (ASL) perfusion MRI to assess CBF in subjects with and without migraine. We found that an incomplete circle of Willis is more common in migraine with aura subjects than controls, and is associated with alterations in cerebral blood flow.

## Methods

### Ethics statement

The study was approved by the University of Pennsylvania Institutional Review Board; all participants provided written informed consent.

### Study population

We performed a prospective case-control study comparing headache-free controls to migraine with aura (MWA) and migraine without aura (MwoA) subjects. Participants were recruited from the neurology clinic at the University of Pennsylvania and by advertisements in the wider community. Eligible participants were 25–50 years old, had a diagnosis of MWA or MwoA using International Classification of Headache Disorders criteria, or were headache-free controls [14]. Participants under age 25 were excluded to minimize misclassification of subjects who might harbor migraine but not yet have become symptomatic; this was based on epidemiologic data suggesting the large majority of subjects who will develop migraine become symptomatic by age 25 [15], [16]. Participants over age 50 were excluded based on prior data showing acquired changes in circle of Willis anatomy in older subjects [11]. Subjects with any history of cerebrovascular or cardiovascular disease or other neurologic illness were excluded. Patients with prior neurovascular imaging studies were excluded to minimize the potential for referral bias. The study aimed to enroll MWA, MwoA, and control subjects in a 1∶1∶1 ratio. Control subject recruitment was targeted to match the migraine cohort by age and sex. Participants were screened and examined by a single study neurologist to ensure that they met inclusion/exclusion criteria, and were enrolled between March 2008 and June 2012.

### Magnetic resonance imaging acquisition

Using 3 Tesla MRI (Siemens Trio, Erlangen, Germany), the following sequences were obtained: T1-weighted localizer scans (TR/TE = 20/5 msec-8 mm slice-28 cm FOV-192x144 matrix-1 NEX), 3D volumetric T1-weighted MPRAGE scan (TR/TE = 1620/3.87 ms-1×1×1 mm^3^ voxel size-25.6 cm FOV-256x256 matrix-1 NEX), and 3D time of flight MRA (TR/TE = 26/3.4 ms-1 mm slice/0.5 mm overlap-25 cm FOV-832x571 matrix). Resting CBF measurements were acquired using pseudocontinuous ASL with gradient-echo echo-planar imaging readout (TR/TE = 4000/18 ms-slice thickness/gap = 6 mm/1.2 mm-voxel resolution = 3.5×3.5×6 mm^3^-labeling duration 1125 ms-post-labeling delay time 1200 ms) [17]. The labeling plane was positioned 90 mm below a 16 slice imaging slab. Thirty pairs of interleaved control and tag images were acquired.

### Circle of Willis analysis

MR angiograms were reviewed by two independent neuroradiologists blinded to clinical data. In cases of disagreement, a third blinded rater reviewed the images, selecting one of the prior raters' scores as the final rating. Both maximum intensity projections and source images were used for circle of Willis classification, which was performed using the scheme described by Krabbe-Hartkamp [11]. In this scheme, each component vessel of the circle is rated as present, hypoplastic, or absent, with vessels ≥0.8 mm diameter considered present and those visible but <0.8 mm considered hypoplastic. Separate template-based patterns for the anterior and posterior portions of the circle were used to categorize variants, with hypoplastic vessels considered absent for template categorization, again consistent with the scheme described by Krabbe-Hartkamp. Overall assessment was then classified as 1) complete (all component vessels of both anterior and posterior parts of the circle visible and ≥0.8 mm diameter) or 2) incomplete (either anterior or posterior parts of the circle incomplete). In addition to circle of Willis assessment, intracranial vertebral artery morphology was also assessed. Vertebral arteries were classified as hypoplastic if the diameter was <2 mm, consistent with prior studies [18]. Subjects with a hypoplastic vertebral artery were scored as having incomplete vertebral artery supply.

The primary pre-specified outcome measure was the proportion of subjects with an incomplete circle of Willis in each group. Pre-specified secondary analyses included comparison between groups of 1) complete versus incomplete anterior/posterior portions of the circle, 2) specific morphologic variants of fetal-type posterior cerebral artery (PCA), in which the PCA arises directly from the terminal internal carotid artery, with or without an intact P1 segment connecting the PCA to the basilar artery, 3) morphology of the intracranial vertebral arteries, and 4) burden of circle of Willis variants.

Analysis for circle of Willis variant burden was performed in two ways. First, a variant burden score was computed by assigning points to each vessel in the circle (0 = present, 1 = hypoplastic, 2 = absent) and summing the total number of points. Second, the number of subjects with ≥2 variants in the individual components of the circle was compared across groups. Given prior reports of a difference in circle of Willis morphology by sex, analysis stratified by sex was also performed [11].

### Perfusion imaging analysis

ASL analysis was performed using ASLtbx [19]. For each subject, raw ASL images were coregistered to the MPRAGE images and label/control images were separately motion-corrected using a 6-parameter rigid body spatial transformation. Pairwise subtraction images were then generated after smoothing with a 6mm full-width half-maximum kernel, and CBF maps were created as has been previously described [20]. The CBF map from each subject was normalized to the Montreal Neurological Institute template, and regional CBF in vascular regions of interest (ROI) were extracted using the SPM Pickatlas utility with a custom template for the vascular territories adapted from published reports [21], [22]. ROI included the territories of the anterior (ACA), middle (MCA), and posterior cerebral arteries, as well as combined ACA+MCA territories (anterior circulation) and the entire hemisphere.

To assess the functional impact of circle of Willis variations on CBF, regional CBF in the anterior circulation and PCA circulation was analyzed based on the presence of circle of Willis variants. In addition, to examine whether an incomplete circle of Willis was associated with hemispheric asymmetries in cerebral perfusion, asymmetry ratios were computed for regional CBF (left ROI– right ROI/ left ROI + right ROI, with a value of 0 representing complete symmetry). Absolute values of the asymmetry ratios were used for comparisons between subjects with complete versus incomplete circle of Willis morphology.

### Sample Size

We estimated that approximately 50% of normal controls would have a complete circle of Willis (age and sex-matched to the primarily young, female migraine population). Using an estimate of 20% of migraine patients with a complete circle, and using a corrected chi-square test with α = 0.05 and β = 0.10 (power = 90%), the estimated sample size was 57 subjects in each group to demonstrate a significant difference between either migraine group and controls. Our target sample size was therefore 175 subjects.

### Statistical analysis

Baseline characteristics were analyzed using the chi-squared or Fisher's exact test for dichotomous or categorical variables and the *t*-test or Wilcoxon ranked-sum tests for continuous variables as appropriate. Interrater reliability of MRA interpretation was determined with kappa statistics. For individual vessels, quadratic-weighted kappa was used based on classification as present/hypoplastic/absent. Unweighted kappa was used to assess overall dichotomous determination of complete vs. incomplete circle of Willis. Dichotomous analyses of circle of Willis variants were performed using the chi-squared test. The circle of Willis burden score was similarly analyzed both dichotomously and as an ordinal scale using the Wilcoxon ranked-sum test. CBF values were analyzed using t-tests and linear regression. Absolute values of the hemispheric asymmetry scores were compared using Wilcoxon ranked-sum tests. An association was considered significant if *p*<0.05. As recommended by biostatistical experts, corrections for multiple comparisons were not performed [23]. Statistical analyses were performed using JMP (Version 9, SAS Institute Inc., Cary, NC) and Stata (Version 10.0, Stata Corp., College Station, TX).

## Results

### Study subjects

A total of 178 subjects were enrolled in the study. Eight subjects were excluded prior to data analysis: 4 could not tolerate MRI due to claustrophobia, 1 had severe MRI artifact from permanent eye makeup, 1 had incorrect MRA field of view setting such that circle of Willis was not visualized, and 2 were incorrectly enrolled despite exclusion criteria. For these latter 2 subjects, one was a control subject who developed new-onset headaches not meeting criteria for migraine between screening and MR scanning (circle of Willis was complete), and one was a MwoA subject with a caudate infarction on MRI who denied history of stroke at screening but subsequently described clinical stroke symptoms years earlier consistent with the infarct topography (circle of Willis was incomplete). 170 participants were included in the final analysis. Subject characteristics are presented in Table 1. There was a trend towards more female subjects in the MWA compared to control group (86% vs. 72%, p = 0.07).

**Table 1:** Clinical characteristics of groups.

	Control (n = 53)	MWA (n = 56)	MwoA (n = 61)	p value MWA vs. control	p value MwoA vs. control
Age (mean±sd)	32.2±6.0	33.6±6.9	33.9±6.9	0.26	0.15
Female, n (%)	38 (72)	48 (86)	46 (75)	0.07	0.65
Systolic blood pressure (mean±sd)	125±17	125±19	128±18	0.86	0.38
Diastolic blood pressure (mean±sd)	80±9	82±12	84±13	0.28	0.05
Hypertension, n (%)	1 (2)	2 (4)	4 (7)	1.0	0.37
Diabetes, n (%)	0 (0)	1 (2)	0 (0)	1.0	1.0
Smoker, current or former, n (%)	11 (21)	13 (23)	25 (41)	0.76	0.03
Oral contraceptive use, current or former (women only), n (%)	32 (84)	37 (77)	42 (91)	0.41	0.32
Migraine duration, years (mean±sd)		18.4±9.8 a	15.2±8.9		
Migraine frequency, per month (median, IQR)		3 (1–5) ^b^	2 (1–6)		

a p = 0.07 vs MwoA; ^b^ p = 0.57 vs MwoA.

Abbreviations: MWA, migraine with aura; MwoA, migraine without aura; sd, standard deviation; IQR, intraquartile range.

### Reliability of MRA interpretation

Interrater reliability was generally substantial for individual vessels, with the exception of the anterior communicating artery, which had only moderate reliability. Kappa values for each segment were as follows: left/right A1 segment of the ACA 0.80/0.72, anterior communicating artery 0.59, left/right P1 segment of the PCA 0.74/0.83, left/right posterior communicating artery 0.69/0.73, left/right vertebral artery 0.75/0.81. For dichotomous total circle of Willis categorization (complete vs. incomplete), there was agreement for 80% of cases and kappa = 0.60; for the anterior portion agreement was 84% and kappa = 0.47, and for the posterior portion agreement was 85% and kappa = 0.71.

### Circle of Willis morphology on MRA

Table 2 presents circle of Willis morphology across the groups. Analysis of the primary pre-specified outcome measure showed a significant difference between groups, with an incomplete circle of Willis more common in MWA subjects compared to controls (73% vs. 51%, p = 0.02), and a similar trend in MwoA subjects (67% vs. 51%, p = 0.08). For the MWA group, a significant difference compared to controls was seen for both anterior and posterior portions of the circle.

**Table 2:** Circle of Willis morphology between groups.

	Control (n = 53)	MWA (n = 56)	MwoA (n = 61)	Migraine combined (n = 117)	*p* value MWA vs. control	*p* value MwoA vs. control	*p* value Migraine vs. control
Overall COW incomplete, n (%)	27 (51)	41 (73)	41 (67)	82 (70)	0.02	0.08	0.02
Anterior portion COW incomplete, n (%)	7 (13)	18 (32)	13 (21)	31 (26)	0.02	0.26	0.05
Posterior portion COW incomplete, n (%)	22 (41)	36 (64)	35 (57)	71 (61)	0.02	0.09	0.02
VAincomplete, n (%)	11 (21)	13 (24) a	10 (16)	23 (20) a	0.72	0.55	0.89
Fetal PCA, n (%)	18 (34)	17 (30)	16 (26)	33 (28)	0.69	0.37	0.45
Isolated fetal PCA (no P1 segment), n (%)	2 (4)	5 (9)	8 (13)	13 (11)	0.27	0.08	0.12

Abbreviations: MWA, migraine with aura; MwoA, migraine without aura; COW, circle of Willis; VA, vertebral arteries; PCA, posterior cerebral artery.

a 1 patient in the MWA group had incomplete imaging of the vertebral arteries.

**Figure 1:**
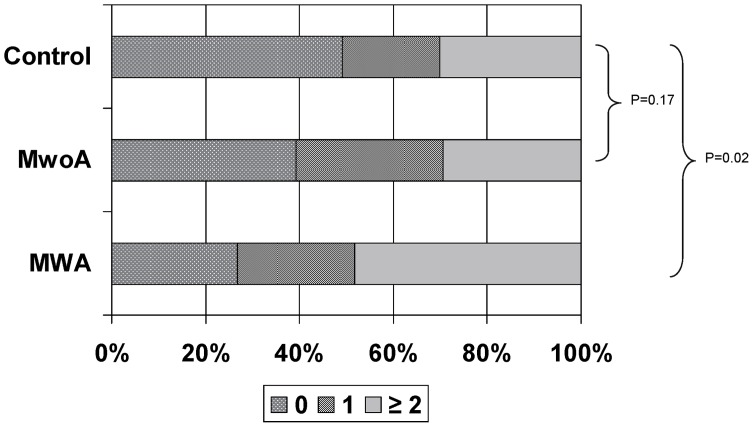
Circle of Willis variant burden scores by group. (MWA, migraine with aura; MwoA, migraine without aura).

The circle of Willis variant burden score was higher in MWA subjects than controls (p = 0.02), but not in MwoA subjects compared to controls (p = 0.17). (Figure 1) More MWA subjects had variant burden scores ≥2 compared to controls (48% vs. 30%, p = 0.05); there was no difference between MwoA and control groups (41% vs 30%, p = 0.23). There was a trend for MWA subjects to be more likely to have ≥2 variants in the individual component vessels of the circle compared to controls (36% vs. 21%, p = 0.08), but no difference between MwoA and controls (16% vs. 21%, p = 0.55).

An incomplete circle of Willis was less common in women compared to men (59% vs. 79%, p = 0.03), with generally similar sex differences regardless of migraine status (Table 3).

**Table 3:** Impact of sex on circle of Willis morphology.

	Anterior portion COW incomplete	p	Posterior portion COW incomplete	P	Overall COW incomplete	P
Overall cohort						
Female (%)	30/132 (23)	0.84	66/132 (50)	0.02	82/132 (60)	0.03
Male (%)	18/38 (21)		27/38 (71)		30/38 (79)	
Controls						
Female (%)	5/38 (13)	0.99	12/38 (32)	0.02	17/38 (45)	0.15
Male (%)	2/15 (13)		10/15 (67)		10/15 (67)	
MWA						
Female (%)	14/48 (29)	0.24	30/48 (62)	0.47	33/48 (69)	0.06
Male (%)	4/8 (50)		6/8 (75)		8/8 (100)	
MwoA						
Female (%)	11/46 (24)	0.38	24/46 (52)	0.15	29/46 (63)	0.22
Male (%)	2/15 (13)		11/15 (73)		12/15 (80)	

Abbreviations: MWA, migraine with aura; MwoA, migraine without aura; COW, circle of Willis.

### Cerebral blood flow and circle of Willis variations

Perfusion MRI was available for 156 subjects; 14 subjects had uninterpretable perfusion imaging due to technical artifact. There was no significant difference in global or regional CBF between groups. Both global and regional CBF were significantly higher in women than men (p<0.001). Analysis of regional CBF divided into the anterior (ACA+MCA) and PCA circulation is presented in Table 4. Regional CBF in the territory of the PCA was significantly lower in subjects with an incomplete posterior portion of the circle (53.3 vs. 58.4 ml/100g/min, p = 0.02). This effect was attenuated in multivariate analysis adjusting for sex (p = 0.11).

**Table 4:** Comparison of mean cerebral blood flow (CBF, ml/100g/min) in different vascular territories by patient characteristics.

	ACA+MCA CBF	p value	PCA CBF	p value
Female	59.3±13.1	<0.001	58.5±13.4	<0.001
Male	50.6±11.4		45.8±11.3	
Control	57.6±12.1	0.99	54.7±13.9	0.86
MWA	57.2±13.1		56.1±14.2	
MwoA	57.3±14.5		56.0±14.1	
Anterior COW complete	57.3±13.7	0.91	54.6±14.4	0.12
Anterior COW incomplete	57.5±11.6		58.8±11.8	
Posterior COW complete	58.3±14.3	0.42	58.4±14.7	0.02
Posterior COW incomplete	56.6±12.3		53.3±13.0	
Fetal PCA – absent	58.1±13.4	0.15	55.8±14.4	0.95
Fetal PCA – present, unilateral	56.6±13.0		55.1±14.0	
Fetal PCA – present, bilateral	48.9±8.4		54.7±8.1	
Isolated fetal PCA (no P1 segment), absent	57.8±13.2	0.17	55.9±14.0	0.30
Isolated fetal PCA (no P1 segment), present	52.5±13.0		51.7±13.2	
VA complete	58.1±12.5	0.27	56.1±13.7	0.46
VA incomplete	54.4±16.0		53.8±15.0	

Abbreviations: ACA, anterior cerebral artery; MCA, middle cerebral artery; PCA, posterior cerebral artery; CBF, cerebral blood flow; MWA, migraine with aura; MwoA, migraine without aura; COW, circle of Willis; VA, vertebral arteries.

Hemispheric CBF was more asymmetric in subjects with an incomplete compared to complete circle of Willis (median hemispheric asymmetry ratio 0.017 vs.0.013, p = 0.05). There were no differences in the hemispheric asymmetry ratio based on anterior or posterior circle status alone. PCA CBF was significantly more asymmetric in subjects with a fetal PCA without P1 segment (median PCA territory asymmetry ratio 0.050 vs. 0.021, p = 0.02), and in those with a unilateral fetal PCA compared to those with no fetal PCA or bilateral fetal PCA (0.034 vs 0.018 vs. 0.014, p<0.001). There was no difference in PCA territory asymmetry based on completeness of the overall circle, anterior or posterior circle subsets, or the vertebral arteries. There was also no difference in anterior circulation asymmetry based on circle of Willis morphology. Analysis showed no significant interaction between migraine status, circle of Willis status, and CBF parameters.

## Discussion

More than 45 years ago, Pearce and Foster first suggested the possibility of an association between migraine and circle of Willis variants based on a small study using catheter angiography [24]. More recently, a hypothesis for how circle of Willis variants might contribute to migraine pathogenesis has been proposed [25]. In brief, circle of Willis variants might contribute to decreased or more variable regional CBF and impair the ability to augment flow in the setting of increased metabolic demand. This dysregulation of CBF might allow relative ischemia to develop, particularly in the setting of increased demand related to the neuronal hyperexcitability seen in subjects with migraine [26]. Ischemia is known to increase susceptibility to cortical spreading depression, which plays a critical role in the genesis of migraine attacks [9], [27]. Given that migraine is a complex disorder, it seems plausible that the combination of developmental abnormalities in the structure of the cerebrovascular circulation, neuronal hyperexcitability, and possible alterations in vascular tone and endothelial function [28], [29], [30] might work synergistically to increase susceptibility to cortical spreading depression and subsequent migraine. This also suggests one possible mechanism contributing to the increased risk of cerebral infarction in migraine subjects.

Two studies using MRA have reported an association between variant circle of Willis and migraine, although these studies have had limitations, including use of lower field strength MRA, control groups consisting of patients with other neurologic diseases undergoing MRA for clinical reasons, and variable methodology for defining circle of Willis variants [31], [32]. Two additional studies have been presented only in brief letter form [33], [34]. Widely different frequencies of variant circle of Willis morphology have been reported in these studies, likely reflecting different methods of defining circle of Willis variants (Table 5). In our study, we used an established classification scheme to define circle of Willis morphology. The frequency of an incomplete circle in our control group was highly consistent with that reported in a previous study in healthy subjects using the identical classification scheme, in which an incomplete anterior portion was found in 14% of younger (age 20–25) and 32% of older (age 60–88) subjects, and an incomplete posterior portion in 38% of younger and 53% of older subjects [11]. Our results, interpreted in conjunction with earlier data, suggest that variations in cerebrovascular anatomy likely do contribute to migraine susceptibility.

**Table 5:** Summary of studies examining circle of Willis morphology and migraine using magnetic resonance angiography.

	Anterior circle of Willis incomplete				Posterior circle of Willis incomplete			
	Control	MWA	MwoA	Migraine combined	Control	MWA	MwoA	Migraine combined
Present study n = 170	13%	32%	21%	26%	41%	64%	57%	61%
Cavestro [32] n = 429	5%	9%	10%	10%	13%	35%	24%	26%
Bugnicort [31] n = 124	5%	9%	4%	6%	18%	61%	38%	49%
Schoonman [33] n = 44	17%	NR	NR	9%	67%	NR	NR	50%
Ikeda [34] n = 173	NR	NR	NR	NR	45%	81%	48%	62%

NR  =  not reported.

The functional and clinical implications of circle of Willis variants have been studied only to a limited degree. In patients with a fetal-type PCA, internal carotid artery flow volume is increased and basilar artery flow decreased, and large asymmetries in internal carotid artery flow are seen in patients with absent ACA A1 segments [12]. The absence of a posterior or anterior communicating artery has been associated with an increased risk of cerebral infarction in patients with carotid stenosis, and the presence of a hypoplastic vertebral artery has been associated with an increased risk of posterior circulation infarction [35], [36], [37], [38]. A fetal-type PCA has been associated with a reduction in deep white matter lesions seen on MRI, suggesting a possible protective effect of this anomaly [39]. Our results support a functional impact of circle of Willis variants on the distribution of resting CBF. We also found that CBF was significantly greater in women, a finding that has been demonstrated previously [40], [41].

Strengths of our study include a prospective design with pre-specified outcome measures, rigorous blinded MRA interpretation using a previously defined classification scheme, a control group free of neurologic disease, and use of high-resolution 3T MRA, which has been shown to significantly improve imaging of the smaller vessels within the circle of Willis compared to lower field strength MRA [42]. The major limitation of our study was the sample size, which limited our power to perform extensive analysis of subgroups of circle of Willis variants, both in terms of their association with migraine, but also in terms of impact on CBF. For the latter measure, an analysis including laterality of circle of Willis variants would be desirable, but was limited by the available sample size. While our study demonstrated an association between circle of Willis variants and certain measures of CBF and a difference in the prevalence of circle of Willis variants between migraine and control groups, overall CBF differences between migraine and control subjects were not found.

There was a modest, non-significant excess of female subjects in the MWA compared to MwoA and control groups. Prior studies of circle of Willis variants have shown a lower prevalence of an incomplete circle in women, a finding also replicated in our study [11]. This would suggest that, if anything, the slight sex imbalance in the MWA group would bias towards showing a smaller difference in circle variants between the MWA and control groups. An additional limitation of our study is the potential misclassification of subjects as headache-free when they might develop migraine later in life. While we attempted to minimize this by excluding subjects under age 25, this is an imperfect solution given that new onset migraine does occur above this age threshold [15], [16]. However, this type of misclassification would also be expected to weaken the ability to show a difference between groups. Subjects for this study were recruited from the neurology clinic and the wider community of a large, urban university with a tertiary care hospital, which may affect the generalizability of the findings to other populations.

In summary, we found an incomplete circle of Willis more common in MWA compared to control subjects, with MwoA subjects having an intermediate prevalence. An incomplete circle of Willis was associated with greater asymmetry in hemispheric CBF and PCA variants were associated with greater PCA territory CBF asymmetries. The identification of circle of Willis variations as a factor associated with migraine might be useful from a diagnostic perspective to help classify migraine patients according to specific factors contributing to their disease, which in turn might eventually have implications for selection of therapeutic strategies. It remains to be determined how circle of Willis variations contribute mechanistically to migraine susceptibility and whether circle of Willis variants are related to the risk of cerebral ischemia in migraine.
